# Nanoparticles for Inducing Antigen-Specific T Cell Tolerance in Autoimmune Diseases

**DOI:** 10.3389/fimmu.2022.864403

**Published:** 2022-03-22

**Authors:** Naomi Benne, Daniëlle ter Braake, Arie Jan Stoppelenburg, Femke Broere

**Affiliations:** ^1^Department of Biomolecular Health Sciences, Faculty of Veterinary Medicine, Utrecht University, Utrecht, Netherlands; ^2^Department of Rheumatology, University Medical Center Utrecht, Utrecht, Netherlands; ^3^Department of Clinical Immunology, University Medical Center Utrecht, Utrecht, Netherlands; ^4^Department of Clinical Sciences, Faculty of Veterinary Medicine, Utrecht University, Utrecht, Netherlands

**Keywords:** nanoparticles, autoimmunity, tolerance, antigen, immunotherapy, Treg

## Abstract

Autoimmune diseases affect many people worldwide. Current treatment modalities focus on the reduction of disease symptoms using anti-inflammatory drugs which can lead to side effects due to systemic immune suppression. Restoration of immune tolerance by down-regulating auto-reactive cells in an antigen-specific manner is currently the “holy grail” for the treatment of autoimmune diseases. A promising strategy is the use of nanoparticles that can deliver antigens to antigen-presenting cells which in turn can enhance antigen-specific regulatory T cells. In this review, we highlight some promising cell targets (e.g. liver sinusoidal endothelial cells and splenic marginal zone macrophages) for exploiting natural immune tolerance processes, and several strategies by which antigen-carrying nanoparticles can target these cells. We also discuss how nanoparticles carrying immunomodulators may be able to activate tolerance in other antigen-presenting cell types. Finally, we discuss some important aspects that must be taken into account when translating data from animal studies to patients.

**Graphical Abstract:**
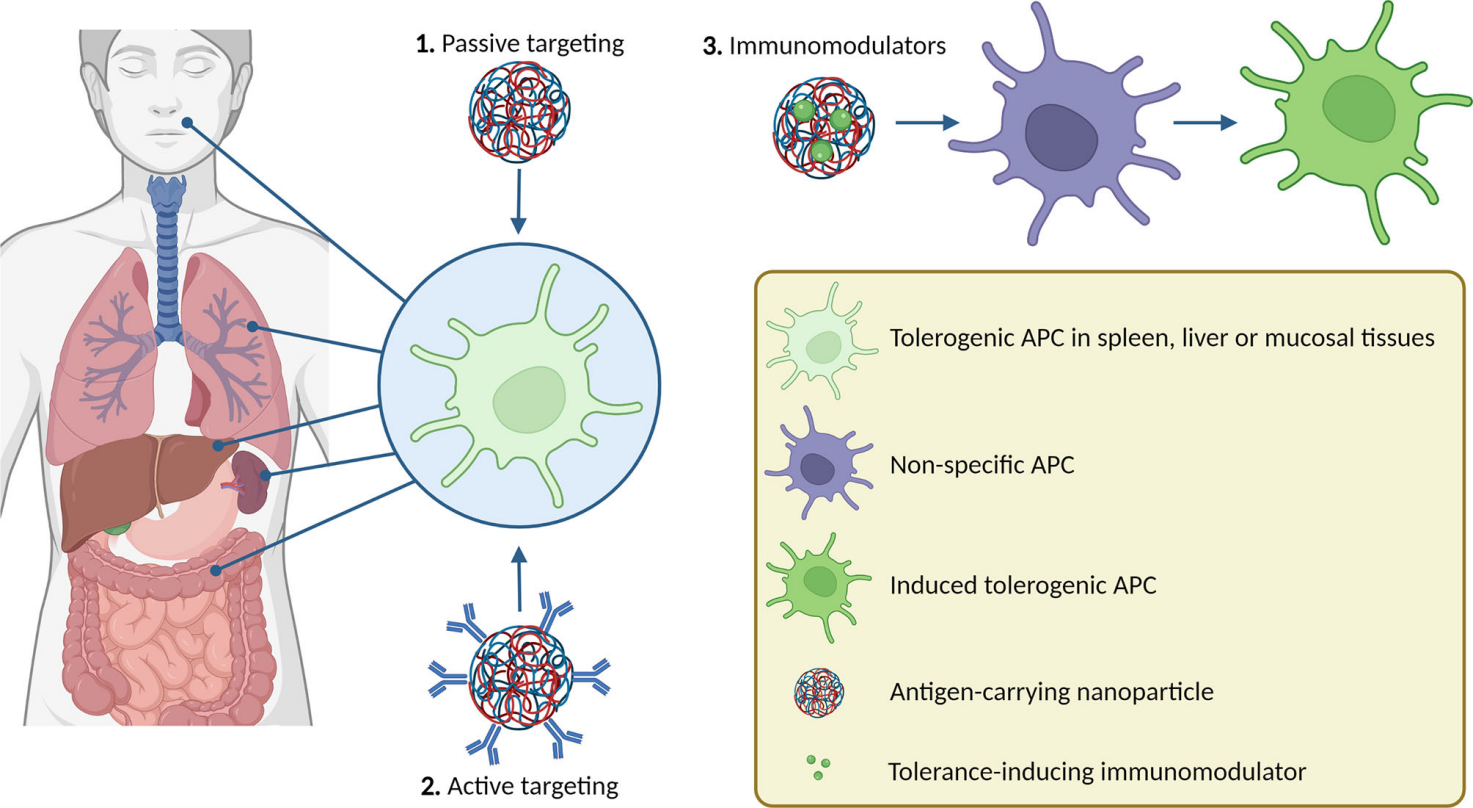


## 1 Introduction

### 1.1 General

Our immune system has evolved to distinguish non-self from self-antigens to protect us against pathogens while also maintaining tissue homeostasis. Immune cells of both the innate and the adaptive immune systems are involved in this complex protection against potentially harmful intruders. The process of combating exogenous antigens by these immune cells is a powerful effector mechanism. However, when directed toward our own cells, tissues, or commensal microbes, this mechanism could be very destructive (1). To maintain immune homeostasis and prevent tissue damage, the immune system must be able to distinguish innocuous endogenous antigens from potentially harmful exogenous antigens (2). This mechanism is known as self-tolerance and is maintained by specialized immune cell subsets such as tolerogenic antigen-presenting cells (APCs) and regulatory T cells (Tregs) (3). In autoimmunity, Tregs are the most well-studied cell type and the focus of this review. Tregs can be derived from the thymus (4) or peripherally induced from effector T cell populations (5). Tregs are typically defined as CD45RO^+^TCR^+^CD4^+^CD25^hi^Foxp3^+^CD127^-^ cells in humans, and CD4^+^CD25^+^Foxp3^+^ in mice (6), but can also express the suppressive receptors CTLA-4, PD-1, TIGIT, and GITR, among others (7, 8). Other suppressive T cell populations that do not express Foxp3 include T regulatory 1 (Tr1) cells, which secrete high levels of IL-10 and express CD49b and LAG-3 in humans and mice (9), and T helper 3 (Th3) cells, which secrete high levels of TGF-β (10). Apart from T cells, B cells also play an important role in maintaining immune homeostasis, including regulatory B cells (Bregs) (11), of which there are also several subtypes, as reviewed elsewhere (12). Immune tolerance can be disrupted due to multifactorial causes (13). This can prompt an overactive inflammatory response towards (auto)antigens (14), resulting in the development of autoimmune diseases. Current treatment modalities focus on the reduction of disease symptoms using anti-inflammatory drugs (e.g. corticosteroids) or biologicals (e.g. tumor necrosis factor (TNF)-α inhibitors) (15). Long-term use of these drugs coincides with unwanted side effects such as increased susceptibility to opportunistic infections and tumors (16). Significant research has focused on improving the targeting of these drugs, thereby reducing the dosage, and limiting off-target effects [as reviewed by Fang et al. (17)]. Although these therapies have improved patient care over the last decades, they fail to cure patients that suffer from autoimmune diseases, necessitating lifelong therapy.

Restoring the immune balance by down-regulating auto-reactive cells and enhancing Treg function is a promising strategy to treat autoimmune diseases. Importantly, this could result in long-lasting medication-free disease remission (18). Unfortunately, injection of free antigen is unlikely to result in sufficient accumulation in the specific cells that can induce tolerance. Besides, many autoantigens are poorly soluble, and due to their size or charge can bring about unwanted immunogenic effects if administered freely (19). A very promising approach is the use of APC-like nanoparticles (20), as reviewed elsewhere (21). Another strategy is to develop nanoparticulate delivery systems to facilitate the delivery of antigens to APCs *in vivo*.

Nanoparticles are drug delivery systems ranging from 1 to 1000 nm in size (22). These particles protect their cargo from enzymatic degradation and can be designed to accommodate for an antigen’s size, charge, and solubility, and (passively) target specific immune cells. This allows for higher therapeutic efficacy, reduces the required dose, and minimizes off-target effects or side effects associated with high doses or the intrinsic properties of the free antigen (23). Nanoparticles can target organs or cells depending on their physicochemical properties (e.g. size, charge, and rigidity) (24–27), or by the incorporation of targeting moieties, such as antibodies (28). Nanoparticles can be made of different materials, including polymers (29), metals (30), lipids (31), proteins (32), or a combination ft he above. Some of these materials have intrinsic immunomodulatory effects (33), which makes them interesting for use in immunotherapy. Furthermore, nanoparticles can carry both an antigen and an immunomodulator to induce tolerogenic phenotypes in APCs *in vivo*. In this review, we highlight some promising cell targets and several strategies by which antigen-carrying nanoparticles, with or without immunomodulators can target these cells.

### 1.2 Cellular Targets

Immune tolerance to circulating antigens is maintained by the spleen and liver (34, 35). These organs are responsible for filtering the blood and contain many specialized cell types that are involved in the clearance of apoptotic cells. This process is termed efferocytosis and is vital for immune homeostasis; the dysregulation of this process has been implicated in several autoimmune diseases (36, 37). Some APC subsets such as plasmacytoid dendritic cells (pDCs) express CD36 and CD61, which are efferocytic scavenger receptors involved in immune regulation (38). Cells expressing these receptors often show a decrease in the surface expression of the co-stimulatory molecules CD40 and CD86 and it has been shown that efferocytosis by DC subsets leads to a suppressive phenotype (39, 40). Furthermore, deficiency in scavenger receptor function has been described to be involved in the development of autoimmunity (41), and activation of the efferocytic receptor MER protected mice against the development of arthritis in collagen-induced arthritis and KRN serum transfer mouse models (42). In a study by Watkins et al., the importance of efferocytosis in immune tolerance was demonstrated by directly targeting antigens towards apoptotic erythrocytes. When these erythrocytes were efferocytosed in the spleen, the antigen was presented in a tolerogenic manner and induced long-lasting antigen-specific T cell anergy (43). In another study, it was shown that human apoptotic cells derived from peripheral blood mononuclear cells have low expression of HLA-DR and CD86, produce the anti-inflammatory cytokines IL-10 and TGF-β, and expressed Fas and caspase-3. In a mixed lymphocyte reaction, the apoptotic cells greatly reduced the proliferation of T cells as compared to non-apoptotic cells and reduced the expression of CD25, CD45RO, and OX40 on proliferated T cells. These apoptotic cells inhibited allogeneic immune responses in humanized non-obese diabetic (NOD)/severe combined immune deficiency (SCID)/γC mice (44). Furthermore, i.v. injection of apoptotic cells that expressed a MOG peptide prevented the development of EAE in mice. This was shown to be due to the accumulation of the apoptotic cells in the splenic marginal zone and antigen-specific T cell unresponsiveness, as measured by reduced proliferation of T cells and reduced production of IFNγ and IL-17 by T cells. Unfortunately, injection of the apoptotic cells after MOG immunization did not affect disease progression (45). Splenectomy abolishes the immune-suppressing effects of apoptotic cell-mimicking liposomes (35). These studies show that when antigen is taken up in the context of efferocytosis, it leads to immune suppression and that the spleen is vital in this process. The splenic marginal zone contains several cell subtypes such as marginal zone macrophages (MZMs), B cells, and DCs (46). For instance, MZMs expressing the macrophage receptor with a collagenous structure (MARCO) (47) and macrophages expressing CD169/sialic acid-binding immunoglobulin-type lectin-1 (Siglec-1) (48) can induce tolerance. Depletion of Siglec-1^+^ macrophages from the spleens in mice susceptible to EAE abrogated the protective effects of MOG-apoptotic cells (45). Siglec-1 is important for cell-cell contact and binds to CD8α^+^ DCs, which suggests that the tolerogenic immune effects of Siglec-1^+^ macrophages may be mediated through marginal zone DCs (49). CD8α^+^CD103^+^ DCs in the marginal zone of the spleen efficiently efferocytose apoptotic cells from the blood, and subsequently migrate to splenic T cells to present antigens in a tolerogenic fashion (50). Indeed, injected apoptotic cells are filtered by CD8α^+^ DCs in WT mice and lead to tolerance, while Siglec-1 depletion leads to uptake by CD8α^-^CD11b^+^ DCs instead (45).

The liver microenvironment also promotes tolerance (51) and human DCs derived from the liver are more suppressive than blood-derived DCs (52). The liver contains a multitude of cells that are inherently suppressive (34). For example, liver sinusoidal epithelial cells (LSECs) have been shown to induce differentiation of T cells to both Foxp3^+^ Tregs and Foxp3^-^LAG3^+^ Tr1 cells (53). Furthermore, Kupffer cells in the liver express low levels of co-stimulatory molecules and suppress T cell responses (54).

Finally, it is well known that oral and mucosal antigen application favors tolerance induction. In a collagen-induced arthritis mouse model, multiple oral administrations of type II collagen significantly reduced the severity of the disease (55), and in a human trial in rheumatoid arthritis patients, oral administration of type II collagen significantly reduced joint swelling and pain (56). Oral tolerance induction is independent of apoptotic pathways as splenectomy does not abrogate oral tolerance (57). The mesenteric lymph nodes are important for oral tolerance induction, as it was found that transplantation of peripheral lymph nodes into the gut mesenteries in mice did not allow for the induction of oral tolerance (58). This points to the importance of the microenvironment created by stromal cells in these lymphoid structures that facilitates tolerance induction. In mice removal of the superficial cervical and internal jugular lymph nodes which drain the nasal mucosa abrogated nasal tolerance induction (59). Specifically, it has been shown that CD11b^+^ DCs are important for oral tolerance in a collagen-induced arthritis model (60).

APCs involved in mucosal tolerance are mainly macrophages and DCs in the lungs (61), along with B cells, DCs, and macrophages in the nasal- and gut-associated lymphoid tissues (62–64). Other DCs involved in nasal and oral tolerance express the inhibitory Fc receptor for IgG IIB (FcγRIIB) (65, 66). FcγRIIB plays a key role in DC uptake, processing, and presentation of antigens (67) and a loss of this receptor has been shown to induce autoimmunity in mice (68). Another group of immune-suppressing mucosal DCs are CD103^+^ DCs, which can induce antigen-specific Foxp3^+^ Tregs (69, 70). Collectively, these studies show that there are several subtypes of APCs in the liver, spleen, and oral and mucosal lymphoid tissues which are specialized to induce tolerance and are therefore attractive for targeting nanoparticles. We will highlight several strategies by which this targeting can be achieved.

## 2 Passive Targeting

### 2.1 Physicochemical Properties

#### 2.1.1 Charge

**Figure 1:**
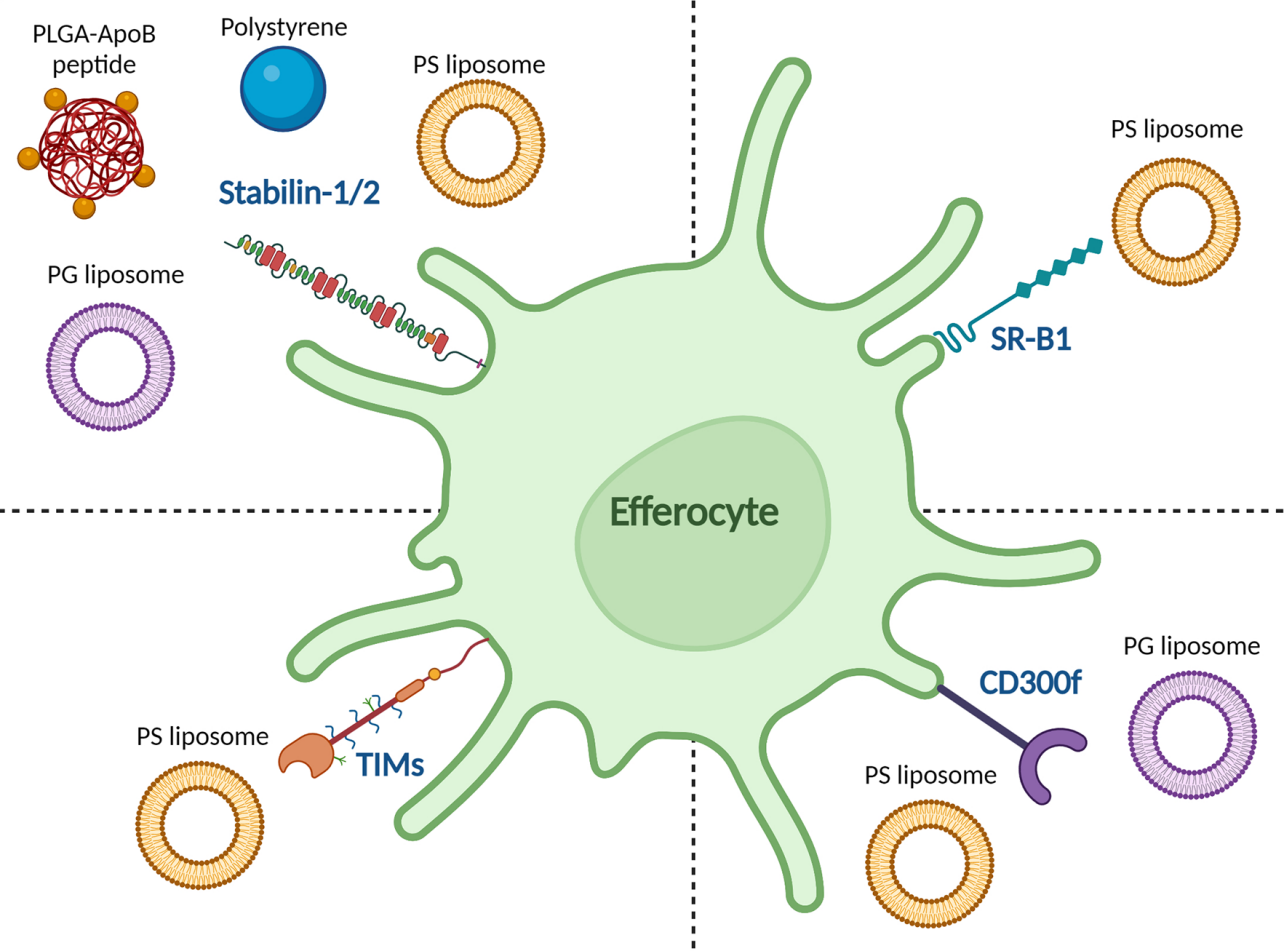
Reported binding of tolerance-inducing nanoparticles to efferocytic receptors on APCs. The stabilin-1/2 receptor was reported to bind to anionic polystyrene beads, apolipoprotein B (ApoB)-peptide-functionalized poly(lactic-co-glycolic acid) (PLGA) nanoparticles, and anionic phosphatidylserine (PS) and phosphatidylglycerol (PG) liposomes. The T cell/transmembrane, immunoglobulin, and mucin (TIM) receptor family recognize PS liposomes as does scavenger receptor class B type 1 (SR-B1). CD300f recognizes both PS and PG liposomes.

One of the great advantages of nanoparticles is that their physicochemical properties can be optimized to the application. Depending on their physicochemical properties, nanoparticles can elicit different immune responses. In the case of pro-inflammatory responses, this is generally achieved by mimicking pathogens (41). However, for tolerance induction, an attractive strategy would be to mimic apoptotic cells. Such apoptotic-like particles would efficiently be taken up directly or *via* the protein corona and processed through the tolerance-promoting effector mechanisms in the spleen and liver. Nanoparticle charge is one of the easiest and most effective ways to achieve this. When cells undergo apoptosis, they express the negatively charged phospholipid phosphatidylserine (PS) on their surface (71), which is recognized by receptors on efferocytes, such as stabilin-2 (72), TIM-4 (73), and CD300f (74, 75), as shown in **Figure 1**. The role of PS in apoptosis is extensively reviewed by Birge et al. and several types of nanoparticles containing PS have taken advantage of this pathway to induce tolerance in autoimmune models (76, 77). Unfortunately, empty PS liposomes have been shown to induce non-specific immune tolerance which may hamper clinical application (35, 78). Anionic phosphatidylglycerol (PG)-containing liposomes encapsulating an atherosclerosis-specific peptide significantly reduced disease progression in an atherosclerotic mouse model. However, the same liposomes without antigen did not have any effect on the disease, meaning that this effect was antigen-specific. The PG-liposomes were more effective at inducing antigen-specific Treg responses than PS-liposomes, even though both had similar surface charges (79). This was hypothesized to be due to the formation of a protein corona, specifically C1q binding to the PG-liposomes, which has been shown to have a tolerogenic effect intricately linked to the clearance of apoptotic cells by binding to scavenger receptors such as class F scavenger receptor (SR-F1) (41, 75, 80, 81). In subsequent studies, it was observed that the liposomes are selectively taken up by APCs in the liver and spleen (unpublished data). In another study, anionic poly(ethylene-co-maleic acid)-poly(lactic-co-glycolic acid) (PEMA-PLGA) nanoparticles encapsulating EAE peptides upregulated PD-L1 expression on liver CD103^+^ DCs and Kupffer cells. This in turn led to antigen-specific lymphocyte unresponsiveness, as measured by reduced proliferation of lymphocytes and reduced production of IL-17, GM-CSF, and IFNγ. The nanoparticles could both prevent and treat EAE in mice. This effect was even observed after splenectomy, underlining the importance of the liver in this model (82). Interestingly, protection against EAE was induced after i.v. injection, and to a lesser extent i.p. injection of nanoparticles, but not after oral or s.c. administration. This was hypothesized to be because these nanoparticles need to travel to the liver and spleen to exert their effects (83).

Splenic macrophages can also be targeted by negatively charged particles (47). PLGA particles coupled to encephalitogenic peptides were localized to the splenic marginal zone after i.v. injection and prevented and treated EAE in mice (84). i.v. injection of polystyrene beads coupled to zinc transporter 8- and islet-specific glucose-6-phosphatase catalytic subunit-related protein-derived peptides could induce antigen-specific tolerance in HLA-A2.1 transgenic mice (HHD) and prevent the development of diabetes in humanized NOD.β2m null HHD mice. The nanoparticles could also suppress antigen-specific CD8^+^ T cell responses in PBMCs from individuals with type 1 diabetes. Specifically, Siglec-1^+^ macrophages and marginal zone DCs produced CCL22 upon nanoparticle uptake, which mediated Foxp3^+^ Treg and CD103^+^ DC chemotaxis *via* CCR4 (85).

LSECs can also be targeted by negatively charged particles since stabilin receptors on LSECs preferentially bind negatively charged particles (86, 87). For instance, Carambia *et al.* designed anionic iron oxide nanoparticles with a poly(maleic anhydride-alt-1-octadecene)-coat. The particles were rapidly cleared from the plasma after i.v. injection and accumulated together with their antigen load in LSECs which led to an increase in Foxp3^+^ Tregs. The nanoparticles induced long-lasting protection to EAE and could even cure EAE in mice. The authors point out that in-depth analysis of the phenotype of nanoparticle-induced Tregs was lacking, as it was not possible to separate nanoparticle-induced Tregs from endogenously induced Tregs (88). Similarly, Saito et al. showed that PEMA-coated polylactide (PLA) nanoparticles accumulated in the liver and spleen after i.v. injection and were mainly associated with Kupffer cells and LSECs in the liver, which showed a reduction in CD86 expression. Ex vivo restimulation of splenocytes with the relevant antigen (PLP_139-151_) resulted in reduced production of IFNγ, IL-17, GM-CSF, TNFα, IL-2 and increased IL-10 in mice immunized with PLP nanoparticles as compared to mice injected with OVA nanoparticles. The PLP nanoparticles prevented EAE in mice (89).

The nanoparticle charge also influences targeting in mucosal tissues. Fromen *et al.* developed anionic and cationic rod-shaped nanoparticles of 80 nm x 320 nm in size, functionalized to ovalbumin. After delivery into the lungs of mice by orotracheal instillation, anionic nanoparticles were immunologically inert, while the cationic particles were pro-inflammatory, as demonstrated by higher gene expression of *Ccl2, Il-10, Il-2, IL-6, Cxcl10, Ifnγ and Il-12β* in homogenized lung cells. This was hypothesized to be because the anionic nanoparticles were taken up by alveolar macrophages, which maintain tissue homeostasis, while the cationic nanoparticles were taken up by lung DCs (90).

#### 2.1.2 Other Physicochemical Properties

Apart from particle charge, the size of the nanoparticles can also determine their biodistribution and uptake by immune cells, as reviewed elsewhere (91). Specifically, concerning targeting tolerance-inducing cell subsets, it was shown that nanoparticles between 79 and 199 nm in size efficiently delivered siRNA to LSECs after i.v. injection, while 420 nm particles did not (92). Particle size is also important for mucosal tolerance induction; i.v. injection of 15 µm, but not 400 nm-sized PLGA nanoparticles led to the retention of these particles in the lungs. While both particles encapsulating PLP were able to prevent EAE development in a mouse model compared to untreated control, the larger particles were more effective (93). In addition, intratracheal administration of glycine-coated polystyrene nanoparticles of 50 nm in size, but not 500 nm were taken up by CD103^+^ DCs in the lungs (26, 94). Finally, 300 nm PLGA nanoparticles encapsulating type II collagen were found in the Peyer’s patches of mice after oral administration. The nanoparticles were able to significantly reduce arthritis scores in a collagen-induced arthritis mouse model. This coincided with reduced circulating anti-type II collagen IgG antibodies, reduced proliferation of draining lymph node lymphocytes after type II collagen restimulation, increased gene expression of *Tgfβ* in Peyer’s patches, and decreased gene expression of *Tnfα* in draining lymph nodes in mice immunized with antigen-loaded nanoparticles compared to placebo (55).

The rigidity of nanoparticles is gaining increasing attention as another important physicochemical parameter for tolerance induction (95). For instance, the FcγRIIB^+^ DCs involved in mucosal tolerance (65, 66) are sensitive to particle rigidity (96). Particle rigidity also influences whether circulating particles can reach sterically obscured cells (97) such as LSECs.

Interestingly, polyethylene glycol (PEG)ylation of PLGA nanoparticles can have profound effects on tolerance induction. Comparing PLGA-PEG to PLGA nanoparticles with identical size and charge, Li et al. show that PEGylation induced lower complement activation, neutrophil recruitment, and co-stimulatory molecule expression on DCs around the injection site after s.c. injection (98).

A unique challenge for the induction of mucosal tolerance is that many antigens are not mucoadhesive. Mucoadhesiveness of antigens is highly dependent on several factors such as hydrophilicity, molecular weight, charge, and chemical structure (99). Nanoparticles designed to be mucoadhesive can overcome this problem, and some of the properties that affect the mucoadhesion of formulations are charge, spreadability/rigidity, and ability to bind to the mucus substrate (100). For example, sublingual administration of ovalbumin adsorbed on mucoadhesive polymerized maltodextrin nanoparticles showed therapeutic tolerance in an ovalbumin-induced allergic mouse model, which was not observed with free ovalbumin. Unfortunately, cellular mechanisms were not reported in this study (101). Pulmonary administration of an EAE antigen with hyaluronic acid [a mucoadhesive (102)] abrogated EAE in mice (103), and peanut-induced anaphylaxis was only inhibited in mice receiving intranasal administration of a nanoemulsion with peanut extract, but not free extract (104). Finally, while only antigen-loaded PLGA nanoparticles increased *Foxp3* gene expression in cervical lymph nodes and suppressed delayed-type hypersensitivity response in mice, intranasal administration of both antigen-loaded PLGA and PLGA-TMC (N-trimethyl chitosan, a mucoadhesive) nanoparticles suppressed proteoglycan-induced arthritis in an antigen-specific manner. The authors hypothesize that the discrepancy between the models could be explained by the chronic nature of the arthritis model compared to the delayed-type hypersensitivity model (105). Collectively, these studies show that the physicochemical properties of nanoparticles can be tuned to induce tolerance by targeting specific organs and/or cell subsets.

## 3 Active Targeting

Aside from targeting cell subsets *via* the physicochemical properties of nanoparticles, particles can be designed to actively target cells *via* the use of targeting moieties (106). The use of targeting antibodies for tolerance induction was extensively reviewed by Castenmiller et al. (28). Functionalization of PLGA nanoparticles with an ApoB peptide (a ligand for the stabilin-1 and stabilin-2 receptors expressed on LSECs) led to higher uptake by LSECs *in vivo* as compared to bare PLGA nanoparticles or mannan (another ligand for LSECs) nanoparticles. i.v. injection of the ApoB nanoparticles induced high TGF-β production by LSECs which coincided with an increase in Foxp3^+^ Tregs in the lungs. The nanoparticles could prevent and treat allergic symptoms in a pulmonary allergen sensitization model in mice (107). Another cell type, CD8^+^CD205^+^ DCs, can induce Foxp3^+^ Tregs from Foxp3^-^ precursors in mice in the presence of a low dose of antigen (108). There is evidence that CD205 is important in the recognition of apoptotic cells (109). Targeting antigens to CD205^+^ cells in the spleen using anti-CD205 antibodies has shown to be effective at deletion of autoreactive CD8^+^ T cells (110), and at inducing tolerance in several autoimmune disease models (111, 112). Targeting Siglec-1 and other C-type lectin receptors can be achieved by glycosylated molecules (113), as reviewed elsewhere (114). Regarding mucosal tolerance, reports of specific targeting of antigens to CD103^+^ DCs using antibodies are divided amongst both pro-and anti-inflammatory responses, and co-administration of a pro-inflammatory adjuvant abrogates the tolerogenic effects of CD103^+^ DCs (115). Targeting FcγRIIB with specific antibodies has been used to induce tolerance (116, 117), but so far these targeting antibodies have not been combined with nanoparticles, although this approach would be promising. Using targeting moieties is an attractive strategy to target APC subsets, but care must be taken to avoid the production of anti-drug antibodies against the targeting ligand (118).

## 4 Immunomodulators

It may not always be necessary to target antigens to a specific APC subset. After systemic injection, APCs rapidly take up nanoparticles. If a nanoparticle encapsulates an antigen together with an immunomodulator, the immunomodulator can direct the APC towards a tolerogenic phenotype. Several studies demonstrate that co-encapsulation of an antigen with an immunomodulator (e.g., rapamycin, calcitriol, aryl hydrocarbon receptor ligands, or NF-κB inhibitors) can ameliorate autoimmunity, whilst free antigen does not (119–129). For example, s.c. injections of nanoparticles encapsulating MOG_35-55_ and dexamethasone significantly treated EAE in mice as compared to empty nanoparticles, dexamethasone nanoparticles, MOG_35-55_ nanoparticles, or free dexamethasone and MOG_35-55_. After ex vivo restimulation of splenocytes with MOG_35-55_ only spleens of mice that received the nanoparticles encapsulating MOG_35-55_ and dexamethasone had reduced IL-17 and GM-CSF production (130). In another study s.c. injection of retinoic acid/TGF-β/insulin peptide-encapsulating PLGA microparticles led to uptake by CD11c^+^ splenic DCs, and a significant increase in B220^+^CD19^+^CD1d^+^CD5^+^ Bregs, but not CD4^+^CD25^+^Foxp3^+^ Tregs in the mesenteric lymph nodes, as compared to control mice. These effects were acute (3 days after particle administration), and it is unknown how long-lasting they are. The particles could prevent diabetes in NOD mice, while the administration of free retinoic acid or TGF-β could not (131). There is also evidence that co-encapsulation is not always necessary. Lewis et al. prepared a mixture of distinct PLGA nano- and microparticles, namely vitamin D3 (1000 nm, phagocytosable), denatured human recombinant insulin (1000 nm), TGF-β1 (30 µm, non-phagocytosable), and GM-CSF (30 µm). When NOD mice were injected s.c. with the mix of particles, the phagocytosable particles were found in the paracortex of the draining lymph nodes and associated mainly with DCs. The particle-positive DCs expressed high PD-L1 and BTLA. In diabetic mice, the particle mixture enhanced Foxp3^+^ Tregs in the spleen and pancreatic lymph nodes and increased PD-1 on CD4^+^ and CD8^+^ T cells. Treatment with all particles administered s.c. significantly prevented and treated diabetes in NOD mice (132). All these studies demonstrate that the immunomodulator enables efficient tolerance induction even without active targeting specific organs or cellular subsets. This is underlined by the fact that these studies reported nanoparticle uptake by multiple subsets of DCs and macrophages in the spleen, lymph nodes, and liver, which gained a tolerogenic phenotype and were able to induce antigen-specific T cell tolerance.

While the current studies are very encouraging it is unclear whether immunomodulators that leak from formulations pose a safety concern by inducing non-specific immune suppression. Luo et al. proposed a nanoparticulate approach that could circumvent this problem. They prepared PLGA-PEG nanoparticles encapsulating the diabetes-specific peptide 2.5mi, together with a CRISPR-Cas9 plasmid and guide RNAs for CD80, CD86, and CD40. Upon i.v. administration, MHC-II^+^CD11c^+^ DCs in the lymph nodes, spleen, and blood took up these nanoparticles, and presented the peptide in the absence of costimulatory molecules, leading to an antigen-specific Foxp3^+^ Treg response. Only the complete formulation significantly reduced diabetes incidence in NOD/Ltj mice (133). In a similar but simpler approach, Krienke et al. made liposomes carrying mRNA coding for disease-relevant antigens that were specifically modified to suppress immune activation. The liposomes were taken up by different splenic CD11c^+^ DCs after i.v. injection. The DCs presented the antigen to T cells in the absence of costimulatory molecules which resulted in antigen-specific Foxp3^+^ Treg expansion and could prevent and treat EAE in mice. Specifically, MOG_35-55_–specific splenic CD4^+^ T cells from mice treated with the liposomes had high expression of inhibitory markers CD5, ICOS, LAG-3, PD-1, CTLA-4, TIGIT, and TIM-3 (134). These studies show that, if the nanoparticle is tolerance-inducing, either by the inclusion of immunomodulators or other methods, it can even skew pro-inflammatory APC subsets towards tolerance.

## 5 Translation to Human

While there are many promising pre-clinical studies with nanoparticles, translation of animal models to human patients is a difficult challenge. It is not fully understood why the translation often fails but can be due to multiple factors. For instance, the protein corona that forms around a nanoparticle after administration can differ between species, which may affect the stability, toxicity, and biodistribution of the nanoparticles (135, 136). Furthermore, there may be differences in phagocytosis of nanoparticles among species; comparing dogs, humans, and several strains of rats and mice, it was found that opsonization of dextran-coated iron oxide nanoparticles occurred mainly *via* the alternative complement pathway in humans, while in the other species this was dependent on Ca^2+^-sensitive pathways (137). Another study tested a wide range of lipid nanoparticles containing mRNA in mice with humanized livers, primatized livers, or “murinized” livers. It was found that mRNA delivery was more efficient to human hepatocytes and primate hepatocytes compared to murine hepatocytes and that there was a discrepancy between the most efficient lipid nanoparticles for delivery to murine vs. human hepatocytes, leading to false positives or negatives. Transcriptomic analysis revealed that in human hepatocytes clathrin-mediated endocytosis was increased while caveolin-mediated endocytosis was decreased after lipid nanoparticle administration, while in mice this was reversed (138). Finally, there are many different models for autoimmunity which have varying degrees of similarity to human patients, which can make the translation to humans difficult (6, 139). For translation from pre-clinical to clinical trials, humanized mice may be useful for studies with nanoparticles, and while some of the studies highlighted in this review use humanized mice (44, 85), this is not standard practice.

To further complicate translation, immune cell number, phenotype, and function can differ between healthy individuals and patients. For example, the DCs in the pancreatic lymph nodes of type I diabetes patients may have reduced tolerogenic function than those of healthy controls, and there were even differences in lymph node cell composition when correcting for sex and age (140). T cells, B cells, DCs, and other lymph node cells are also shown to be different in rheumatoid arthritis patients compared to healthy individuals (141–143). Furthermore, efferocytosis can be defective in patients with autoimmune diseases (144, 145) or obese individuals (146), so for these patients nanoparticles that aim to exploit efferocytosis may not be as effective. The tolerance-inducing strategy could be tailored towards each patient individually if the immune cell function of the patient was matched to the pathway by which the nanoparticles exert their immunomodulatory effects. Encouragingly, one study directly compared nanoparticles with a targeted approach (targeting to LSECs) to a general immune-suppressing approach (rapamycin nanoparticles) in a murine airway inflammation model, and found that both approaches similarly reduced antigen-specific allergic responses (147).

Despite all of these challenges, there have been several successful phase 1 and phase 2 clinical trials using antigen-carrying nanoparticles. Lutterotti et al. coupled myelin-derived peptides to apoptotic PBMCs derived from MS patients. In this phase 1 trial, the cells were well-tolerated, and at a dose >1 x 10^9^ cells there was a decrease in T cell proliferation in response to restimulation with the peptides (148). Another phase 1 clinical trial used CD206-targeting mannosylated liposomes carrying myelin-derived peptides for the treatment of MS. The liposomes were well-tolerated, and patients showed decreased serum concentrations of IL-7, IL-2, CCL2, and CCL4, while TNFα increased (149, 150). Finally, PLGA nanoparticles encapsulating gluten protein were tested in phase 1 and phase 2a trials in patients with celiac disease. The nanoparticles were well-tolerated and significantly reduced antigen-specific IFNγ production. After oral gluten challenge, the patients that received the nanoparticles showed reduced percentages of circulating CD4^+^CD38^+^α4β7^+^, CD8^+^CD38^+^αEβ7^+^, and γδ^+^CD38^+^αEβ7^+^ T cells compared to patients that received placebo (151). These promising results show that there are good prospects for antigen-carrying nanoparticles to treat autoimmunity in human patients.

## 6 Discussion

Current treatments of autoimmune diseases are focused on the management of symptoms using non-antigen-specific anti-inflammatory drugs. These drugs require life-long adherence and can be expensive, especially in the case of the biologicals, while potentially causing severe side effects (152). So far there is no cure for such diseases. Therefore, there is an urgent need to develop antigen-specific treatments for patients, which can induce long-lasting immune tolerance without causing general immune suppression. In this review, we have highlighted different strategies in which nanoparticles can be used to induce antigen-specific tolerance to treat autoimmune diseases and summarized these in **Table 1**. The most commonly used nanoparticles for passive targeting of antigens to APCs are polymeric nanoparticles and liposomes. The most well-studied biodegradable polymeric particles are (PEGylated) PLGA nanoparticles. Several promising studies also use PEMA coating to enhance the efferocytic effects. In studies that prevent and/or treat autoimmunity, the polymeric nanoparticles are anionic and generally around 300 nm in size. For liposomal nanoparticles that carry antigens, again anionic charge, provided by e.g. PG or PS is necessary for tolerance induction. The liposomes further generally contain the zwitterionic helper lipid PC and may contain cholesterol. They can range in size from around 150 nm up to 700 nm. On the other hand, mRNA requires cationic lipids for complexation, so for mRNA delivery, cationic lipids such as DOTMA and DOTAP are commonly used.

**Table 1:** Overview of antigen-carrying nanoparticles that resulted in immune suppression in autoimmune disease models.

Composition	Type	Physicochemical properties	Antigen	Immunomodulator	Targeting molecule	Route	Disease model	Ref
Soybean oil, cetylpyridinium chloride	Nanoemulsion	350-400 nm	Peanut extract	–	–	i.n.	C3H/HeJ mice sensitized to peanut	(104)
PLGA	Polymeric	320 nm, -48.2 mV	Hsp70-mB29a	–	–	i.n.	BALB/c mice with proteoglycan-induced arthritis	(105)
PLGA-N-trimethyl chitosan	Polymeric	448 nm, 24.5 mV	Hsp70-mB29a	–	–	i.n.	BALB/c mice with proteoglycan-induced arthritis	(105)
DOPS^a^, DLPC^b^ and cholesterol	Liposome	628 to 712 nm, -44.9 to -46.6 mV	Insulin_90–110_ (A chain) and Insulin_25–54_ (B chain)	–	–	i.p.	Diabetes-prone NOD mice	(77)
DOTMA^c^ and DOPE^d^	Liposome	300 nm, -30 mV	MOG_35-55_-encoding mRNA	–	–	i.v.	C57BL/6 mice with MOG_35-55_–induced EAE	(134)
DSPC^e^, DSPG^f^ and cholesterol	Liposome	168.9 nm, -55.9 mV	ApoB100_3500-3514_	–	–	i.p.	LDLr-/- mice with western-type diet-induced atherosclerosis	(79)
PLA-PEMA	Polymeric	443.2 nm, -40.2 mV	PLP_139-151_	–	–	i.v.	SJL/J mice with PLP_139-151_-induced EAE	(89)
PLGA	Polymeric	397.5 to 605 nm, -38 to -42.8 mV	PLP_139-151_	–	–	i.v.	SJL/J mice with PLP_139-151_-induced EAE	(27, 84)
PLGA-PEMA	Polymeric	377.9 to 695.6 nm, -46.9 to -72.7 mV	Several peptides/proteins	–	–	i.v.	Diabetes-prone NOD mice, C57BL/6 mice with gliadin-induced celiac disease, SJL/J mice with PLP_139-151_-induced EAE	(82, 83, 153, 154)
Polystyrene	Polymeric	500 nm, anionic	A mixture of HLAA*02:01-restricted epitopes	–	–	i.v.	Humanized diabetes-prone NOD.β2m-deficient HHD mice	(85)
PLGA	Polymeric	299.7 nm, anionic	Type II collagen	–	–	oral	DBA/1 mice with collagen-induced arthritis	(55)
LysoPS^g^ and DMPC^h^	Liposome	169.7 nm, -14.96 mV	FVIII protein	–	–	s.c.	Hemophilia A mice	(155)
PLGA-PEG	Polymeric	286 nm, -23.2 mV	MOG_35-55_	–	–	s.c.	C57BL/6 mice with MOG35-55–induced EAE	(98)
Maltodextrin	Polysaccharidic	60 nm, cationic	Ovalbumin	–	–	sublingual	Ovalbumin-sensitized Balb/c mice	(101)
PLGA	Polymeric	270 nm,-8.63 to -4.56 mV	OVA_323-339_	–	ApoB peptide	i.v.	C57BL/6 mice with OVA-induced allergy	(107)
Hyaluronic acid	Polysaccharidic	3 to 10 nm, anionic	PLP_139-151_	–	LABL peptide	pulmonary	SJL/J mice with PLP_139-151_-induced EAE	(103)
Superparamagnetic iron oxide core with poly(maleic anhydride-*alt*-1-octadecene) coat	Metal	10 nm, -61.6 mV	MBP or MOG peptide	–	poly(maleic anhydride-alt-1-octadecene)	i.v.	B10.PL and tg4 mice with MBP peptide-induced EAE and C57BL/6 mice with MOG-induced EAE	(88)
L-α-egg phosphatidylcholine and L-α-egg phosphatidylglycerol	Liposome	105 nm, -55 mV	Several peptides	Vitamin D3	–	s.c. and i.v.	BALB/c mice with proteoglycan-induced arthritis or HLA-DR15–transgenic, MHC class II^–/–^Fcgr2b^–/–^ mice with α3_135–145_-induced autoimmune Goodpasture’s vasculitis, Diabetes-prone NOD mice	(125, 126)
Acetalated dextran	Polysaccharidic	111 to 127 nm	MOG_35-55_	Dexamethasone	–	s.c.	C57Bl/6 mice with MOG_35-55_-induced EAE	(130)
PEG-Gold	Metal	60 nm	MOG_35-55_	ITE	–	i.p. and i.v.	C57BL/6 mice with MOG_35-55_-induced EAE, diabetes-prone NOD mice	(123, 124)
Egg phosphatidylcholine	Liposome	Not reported	mBSA	NF-kB inhibitor	–	s.c.	C57BL/6 mice with mBSA-induced arthritis	(122)
PLGA-PEG and N,N-bis(2-hydroxyethyl)-N-methyl-N-(2-cholesteryoxycarbonyl-aminoethyl)ammonium bromide	Lipid-assisted polymeric	138 nm, 23 mV	2.5mi	pCas9 and gRNAs targeting CD80, CD86, and CD40	–	i.v.	Diabetes-prone NOD mice	(133)
Calcium phosphate and dioleoylphosphatydic acid nanoparticles coated with DOPE-PEG, DOPC^i^, and cholesterol	Lipid-coated calcium phosphate	180 nm, -6 mV	Citrullinated peptides derived from type II collagen, fibrinogen, vimentin, and fibronectin	Rapamycin	–	i.v.	Wistar rats with collagen-induced arthritis	(129)
PLGA and PLA-PEG	Polymeric	Not reported	Several peptides/proteins	Rapamycin	–	i.v.	SJL/J mice with PLP_139-151_-induced EAE, BALB/c mice with OVA-induced allergy, and FVIII^-/-^ hemophilic mice	(119–121)
Surface nickel-formulated PLGA	Polymeric	1088.6 nm	Insulin B_9-23_	Retinoic acid, TGF-β	–	s.c.	Diabetes-prone NOD mice	(131)
PLGA	Polymeric	30 μm and 1 μm	Denatured insulin	Vitamin D3, TGF-β1, GM-CSF	–	s.c.	Diabetes-prone NOD mice	(132)

a 1,2-dioleoyl-sn-glycero-3-phospho-l-serine.

b 1,2-didodecanoyl-sn-glycero-3-phosphocholine.

c 1,2-di-O-octadecenyl-3-trimethylammonium propane.

d 1,2-dioleoyl-sn-glycero-3-phosphoethanolamine.

e 1,2-distearoyl-sn-glycero-3-phosphocholine.

f 1,2-distearoyl-sn-glycero-3-phosphoglycerol.

g Lyso-phosphatidylserine.

h Dimyristoyl phosphatidylcholine.

i 1,2-dioleoyl-sn-glycero-3-phosphocholine.

Once a targeting molecule is included in the formulation, the physicochemical properties of the nanoparticles may be less important. Similarly, for nanoparticles that co-encapsulate antigens and immunomodulators, there is a wider range of materials and physicochemical properties that can be used, as targeting specific APC subsets is not the goal. Here, the main focus is on the stability of the particle and the successful encapsulation and retention of the cargo inside the particle.

There have been reports of tolerance induction using antigen-free nanoparticles (35, 78, 94), which can be unfavorable if it leads to general immune suppression. Furthermore, while the incorporation of immunomodulators into nanoparticles is effective, care should be taken that the immunomodulator does not leak from the nanoparticle before reaching the site of action. Therefore, nanoparticle immunotherapy should always be tested for non-antigen-specific effects, and in the case of co-encapsulation of an immunomodulator, *in vivo* stability of the immunomodulator should be evaluated. In the case of using targeting ligands on the surface of nanoparticles, there is a risk for the production of anti-drug antibodies against the targeting ligand, which can abrogate the targeting effects of the nanoparticle, or in severe cases even lead to an unwanted immune response (118). In this case, the prevention of the production of such antibodies e.g. by optimal dosing schemes needs to be taken into account (156).

**Figure 2:**
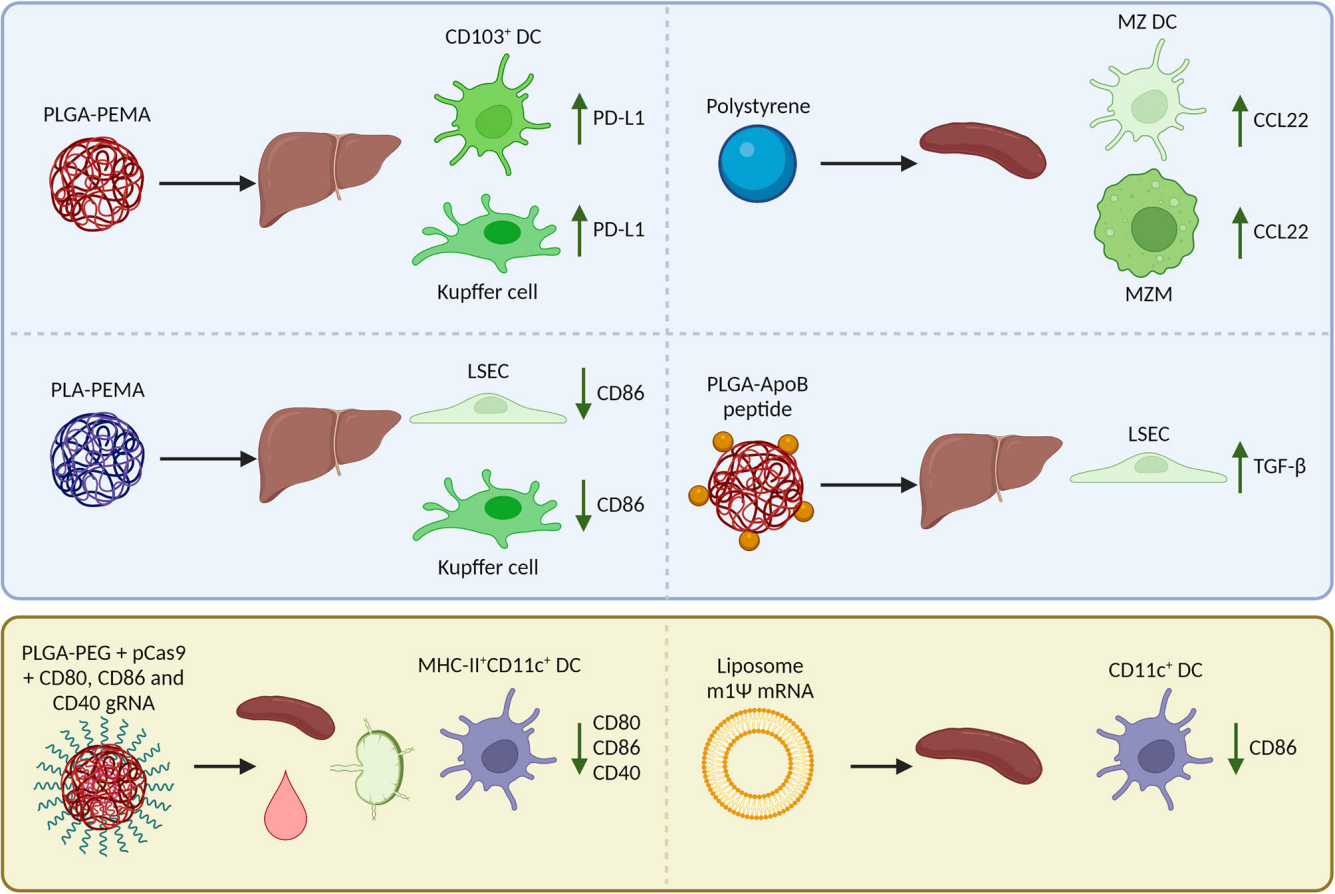
Reported biodistribution, cellular uptake, and Foxp3^+^ regulatory T cell (Treg)-inducing mechanisms in mice after i.v. injection of nanoparticles encapsulating an antigen (peptide/mRNA). The blue box (top) summarizes nanoparticles that target natural tolerogenic antigen-presenting cells in the liver and spleen including dendritic cells (DCs), macrophages, and liver sinusoidal endothelial cells (LSECs) to enhance tolerance. After uptake of nanoparticles, these cells upregulate inhibitory receptors, downregulate costimulatory molecules, produce chemokines that attract Tregs, or cytokines that induce Tregs. The yellow box (bottom) summarizes nanoparticles that are taken up by a wide range of APCs including DCs in the spleen, lymph nodes, and blood. The nanoparticles are designed to inhibit co-stimulation in target cells so that the antigen can be presented in a tolerogenic manner and induce Tregs. PLGA, poly (lactic-co-glycolic acid); PLA, polylactic acid; PEMA, poly(ethylene-co-maleic acid); ApoB, apolipoprotein B; PEG, polyethylene glycol; MZ, marginal zone; m1Ψ mRNA, 1 methylpseudouridine-modified messenger RNA; PD-L1, programmed death-ligand 1; CCL22, C-C motif chemokine 22; TGF-β, Transforming growth factor β.

Where reported in *in vivo* studies, we have outlined the biodistribution of nanoparticles, uptake by APC subsets, and immunological mechanisms for tolerance induction (summarized in **Figure 2**). However, not all studies have examined these aspects, and there is still much to be gained from research that describes well-characterized nanoparticles and their effects on the uptake by APC subsets and subsequent induced immune responses.

## Author Contributions

The manuscript was written by NB with extensive input and editing by all other authors. All authors contributed to the article and approved the submitted version.

## Funding

This work was funded in part by the DC4Balance collaboration project. which is co-funded by the PPP Allowance made available by Health~Holland, Top Sector Life Sciences and Health, to the Dutch Cooperation of Health Foundations (SGF) and the Dutch Arthritis Foundation.

## Conflict of Interest

The authors declare that the research was conducted in the absence of any commercial or financial relationships that could be construed as a potential conflict of interest.

## Publisher’s Note

All claims expressed in this article are solely those of the authors and do not necessarily represent those of their affiliated organizations, or those of the publisher, the editors and the reviewers. Any product that may be evaluated in this article, or claim that may be made by its manufacturer, is not guaranteed or endorsed by the publisher.
